# TRIPS, the Doha Declaration and increasing access to medicines: policy options for Ghana

**DOI:** 10.1186/1744-8603-1-17

**Published:** 2005-12-09

**Authors:** JC Cohen, M Gyansa-Lutterodt, K Torpey, LC Esmail, G Kurokawa

**Affiliations:** 1 Assistant Professor, Leslie Dan Faculty of Pharmacy, University of Toronto; Director, Comparative Program on Health and Society, Munk Centre for International Studies, University of Toronto; 2 Programme Manager, Ghana National Drug Programme, Ministry of Health, Republic of Ghana; 3 Senior Clinical Officer, Family Health International; 4 Ph.D. student, Leslie Dan Faculty of Pharmaceutical Sciences, University of Toronto; 5 Law student, Faculty of Law, University of Toronto

## Abstract

There are acute disparities in pharmaceutical access between developing and industrialized countries. Developing countries make up approximately 80% of the world's population but only represent approximately 20% of global pharmaceutical consumption. Among the many barriers to drug access are the potential consequences of the Trade Related Aspects of Intellectual Property Rights (TRIPS) Agreement. Many developing countries have recently modified their patent laws to conform to the TRIPS standards, given the 2005 deadline for developing countries. Safeguards to protect public health have been incorporated into the TRIPS Agreement; however, in practice governments may be reluctant to exercise such rights given concern about the international trade and political ramifications. The Doha Declaration and the recent Decision on the Implementation of Paragraph 6 of the Doha Declaration on the TRIPS Agreement and Public Health may provide more freedom for developing countries in using these safeguards. This paper focuses on Ghana, a developing country that recently changed its patent laws to conform to TRIPS standards. We examine Ghana's patent law changes in the context of the Doha Declaration and assess their meaning for access to drugs of its population. We discuss new and existing barriers, as well as possible solutions, to provide policy-makers with lessons learned from the Ghanaian experience.

## Introduction

The disparity in pharmaceutical access between developed and developing countries is stark. Developing countries make up approximately 80% of the world's population but only represent approximately 20% of global pharmaceutical consumption[1]. Market failures, government failures and income differences account for this persisting inequity[2]. Specifically, high drug costs, weak or corrupt institutions, contributing to less than effective pharmaceutical purchasing and distribution systems, and the potential consequences of the Trade Related Aspects of Intellectual Property (TRIPS) Agreement all constrain drug access.

Many developing countries have recently modified their patent laws to conform to TRIPS standards, raising the urgency of TRIPS' potentially detrimental impact on drug supply and access. However, recent developments such as the *Decision on the Implementation of Paragraph 6 of the Doha Declaration on the TRIPS Agreement and Public Health *may offer developing countries more freedom to use TRIPS safeguards to address public health needs.

This article focuses on Ghana, a developing country that recently changed its patent laws to conform to TRIPS standards. While Ghana has made strides in improving public health, the country has urgent and serious health needs that cannot be met by the existing system. Improving pharmaceutical access is one of the core challenges facing the Government. As such, there is a menu of choices available for possible use. These include "Paragraph 6", compulsory licensing, parallel importing and attracting investment for the local production of essential medicines to combat HIV/AIDS, malaria and tuberculosis. In this paper, we discuss a selection of pharmaceutical policy choices available to governments that may lead to improved access to medicines. We do this specifically through the case of Ghana. As a test case, Ghana represents the dilemma faced by many developing countries -"make or buy." That is to say, should a government invest more in local production or continue to import medicines? In short, we examine Ghana's patent law changes in the context of the Doha Declaration and assess their meaning for access to drugs of its population. New and existing barriers will be discussed and options for addressing them proposed, to provide policy-makers with lessons learned from the Ghanaian experience.

This paper is based on research conducted for the UK Department for International Development (DfID)[3]. Our study implicitly focuses on the basic pharmaceutical public policy issues that currently face decision-makers in Ghana: 1) how to improve pharmaceutical coverage for the majority of Ghanaians and most importantly for the poor; 2) how to make treatment and drugs more affordable; and 3) how to ensure the government gets maximum value from its pharmaceutical budget. We collected data through: a review of "hard" documents (recent academic literature, policy documents, recent country studies, laws and regulations relating to drug access issues in Ghana and internationally), interviews with key stakeholders and informants to identify major gaps in drug access in Ghana, and inputs from the Access to Medicines Ghana Initiative Advisory Group.

We organize our paper as follows: first, we describe Ghana and its pharmaceutical system; second, we discuss the TRIPS Agreement and the Doha Declaration; third, we discuss Ghana's 1992 Patent Law and 2003 Patent Act; fourth, we discuss Ghana's new Patent Act in the context of the Doha Declaration and of pharmaceutical access in Ghana, followed by conclusions.

### The State of Pharmaceuticals in Ghana

Ghana has made significant improvements in its overall health status over the past few decades, with life expectancy reaching 57 years in 2002 and infant mortality declining to 56 per 1000 live births. Despite these improvements, there are significant health issues facing the country: approximately 3.6% of the population is infected with HIV/AIDS, malaria accounts for 40% of outpatient visits and 25% of mortality under the age of five and the annual risk of tuberculosis infection is approximately 1–2%[4]. High mortality rates, frequent epidemics, unequal access to health services, and uneven health outcomes throughout the country are also major problems.

Pharmaceuticals are available in many health facilities across the country; however, access is largely limited due to financial barriers for most of the population, particularly the poor. According to the World Bank, Ghana had a per capita income of US$380 (2004), which is about one-fifth below the average of US$490 for sub-Saharan Africa. Moreover, a recent study indicates that 40 percent of Ghana's population earns less than minimum wage with this proportion increasing in rural areas[5]. As a result, the poverty level makes it difficult for patients to purchase drugs. For example, HIV/AIDS patients receiving one-month of anti-retroviral therapy paid for by the Global Fund are still required to pay 10% of the costs of medicines, at approximately 50,000 cedis (over $US 5). In real terms, it would require a person who is earning the minimum wage more than 5 working days to cover the co-payment. In a small random sample of interviews with patients at the HIV/AIDS Clinic in St. Martin's Hospital, Agomanya, we observed that many patients were not working at all and had to borrow money from family members to cover this co-payment.

Until recently, Ghana's public health and pharmaceutical system operated under the "cash & carry" (C&C) model, which assumes that drug co-payments can help finance and, therefore, improve the delivery of primary health care services. This system involved a series of self-financing revolving drug funds (RDF), which cascaded down each institutional level, marking up the basic purchase price for drug products to obtain revenue to re-supply the products. Additively, these mark-ups could also increase the price of a drug well beyond the reach of most Ghanaians. The government provided exemptions for co-payments for specific categories, including TB patients, psychiatric patients, children under five years of age, the indigent, pregnant women and the elderly. Identifying who is truly indigent is difficult to do in Ghana because poverty is viewed from a socio-cultural point of view as "shameful" and many poor people are reluctant to admit it. Essentially, this system did not achieve its intent to provide widespread affordable access to medicines for the population.

The Government of Ghana declared its intention to abolish the C&C system and passed a National Health Insurance Bill in 2003, which recently has been implemented. Several districts have also introduced health insurance in their localities. These insurance measures may serve to improve access to medicines; however, their effectiveness is yet to be observed. Importantly, the draft minimum package for pharmaceuticals includes medications for outpatient and inpatient services. Antiretrovirals are covered under different arrangements using the Global Fund to fight AIDS, Tuberculosis and Malaria. The Ministry of Health is reviewing exemption policies and the proposed National Health Insurance drug list to ensure consistency with the Essential Drug List. Despite the difficulties noted above, the Insurance system hopes to use local structures to identify who is "poor" to ensure these categories can access care. A national pricing policy, informed by a comprehensive examination of pharmaceutical pricing models internationally, can also facilitate better financial access of the population to medicines.

Pharmaceutical mark-ups are another policy issue that needs reform. The international research-based and generic pharmaceutical industries provide discounted medicines to Ghana, however once products arrive in Ghana, mark-ups between 11% to 275% wipe out many price advantages[3]. Tax and tariff rates vary but are applicable to all medicines, except public sector procurement done according to the Essential Drug List. In the private sector, depending on the local agent or manufacturer, the cost of antiretrovirals can exceed 32.5% more than the discounted price obtained through the Accelerated Access Initiative (AAI). In some cases, the private health facility adds further margins to increase the cost. In order to effectively address mark-ups, national tax, tariff and mark-up policies need to be reviewed to determine what policy changes could facilitate more affordable prices. Government can regulate wholesale and retail mark-ups on essential medicines. Policies regarding the private sector management chain and public sector supply management chain need examination and adjustment to make medicines more affordable for patients.

Ghana has potential to supply more of its medicine needs; local manufacturing accounts for 20% of the pharmaceutical market share. There are about 30 pharmaceutical manufacturing facilities in Ghana and about 17–18 produce all year round. Most raw materials needed for local manufacture are imported and subject to duties, taxes and tariffs, which erode the potential cost advantage that local manufacturing can provide. Currently, manufacturers pay 12.5% VAT (Value Added Tax), 0.5% EDIF (Economic Development Investment Fund), 0.5% ECOWAS levy, handling and inspection charges, GCNet charges (0.004% of cost and freight)[3]. However, Schedule 1A of the 34 materials of *Active Ingredients of Essential Medicines *are exempt from tax. Manufacturers apply a mark up that can range from 10 to 40%. Wholesalers add a further 10 to 20% when selling to the retailer. Then, the retailer adds another margin of 20 to 50%. All of these margins obviously increase the price of the drug for the patient, thereby contributing to the financial barrier to medicines. To help local industry, the government restricts the importation of 17 pharmaceutical products (e.g. paracetamol, chloroquine). Local production is beneficial given that it also provides employment for the population; however the barrier of limited human and technological capacity must first be overcome.

### The TRIPS Agreement and the Doha Declaration

By way of brief background, the TRIPS Agreement provides minimum standards for intellectual property law and procedures and remedies that should be available so rights holders can enforce their rights effectively[6]. The default principle concerning patents is that they should be available for any invention, whether product or process, in all fields of technology without discrimination[6]. With respect to pharmaceutical patents, the minimum TRIPS obligations include 20 years of patent protection from the inventor's filing date (Article 33), patent rights free of discrimination against the origin of invention or production (Article 27.1), and exclusive marketing rights for the entire patent duration (Article 28)[7]. Transitional periods are granted before TRIPS requirements for patent protection must be met; the deadline for least-developing country members was ultimately extended to 2016 (Articles 65 and 66)[6].

The TRIPS Agreement also outlines provisions around patent rights for member states. For example, Articles 8.2, 31(k) and 40 offer flexibility to member countries to prevent or remedy anti-competitive practice[8]. Article 30 facilitates an early working provision, allowing the limited use of an invention without the patent holder's authorization[9]. Generic companies can use this provision to obtain product approval, facilitating immediate entry into the market after patent expiration. Article 31 permits a government to issue a compulsory license to a third party without the patent holder's consent, if justified in the public interest[7]. Compulsory licensing allows governments to pursue the local production of medicines as one strategy to improve access of the population to essential medicines. Parallel importing, legally pursuant to TRIPS Article 8.1 and Article 6, is the import and resale in a state without the consent of a patent holder, of a patented product in another market. Its rationale is to allow governments and others to "price shop" internationally for pharmaceutical products, based on the underlying principle that the patent holder has been rewarded through the first sale and thus has "exhausted" rights. Compulsory licensing and parallel importation are the focus of this paper.

In practice, governments may realistically be reluctant to exercise TRIPS provisions given some concern about political and economic ramifications, particularly in the area of trade sanctions. The Doha Declaration, issued by the WTO in November 2001, partially aimed to address this concern. It states " [the] TRIPS Agreement *does not *and *should not *prevent members from taking measures to protect public health... [and it] should be interpreted and implemented in a manner supportive of WTO members' right to protect public health and, in particular, to promote access to medicines for all" [emphasis added][10]. Furthermore, the Declaration specifically reaffirms member countries' rights to determine the grounds on which compulsory licenses may be issued, to determine what constitutes a national emergency or circumstance of extreme urgency, and to determine their own regime for the exhaustion of intellectual property rights.

The WTO has, however, not explicitly defined the legal status of the Declaration. An unofficial explanation of the Declaration, available on the WTO website, states that the Declaration provides "important guidance" to individual members and WTO dispute settlement bodies in the interpretation of TRIPS[11]. Correa identifies the Declaration as a "strong political statement" and "a 'subsequent agreement' between the parties regarding the interpretation of a treaty or the application of its provisions, under Article 31.3(a) of the Vienna Convention on the Law of the Treaties"[12]. Ultimately, the functional application of the Declaration is to interpret TRIPS[13], however as Reichman and Hasenzahl note[14], the exact legal status of the Declaration will not be clear until its practical application has been observed through future WTO panels and the Appellate Body.

The Declaration was partially an effort to interpret Article 31(f) of the TRIPS Agreement, which states that compulsory licensing shall be "predominantly for the supply of the domestic market." Given that the majority of developing countries lack the domestic capacity or technical expertise to manufacture on-patent pharmaceuticals, the interpretation of this terminology is crucial for ensuring access to medicine for the poor in many developing countries. On August 30, 2003, the WTO issued a temporary solution, the "Decision on the Implementation of Paragraph 6 of the Doha Declaration on TRIPS Agreement and Public Health"[15]. The Decision temporarily waives Article 31(f), permitting countries with local manufacturing capacity to issue compulsory licenses to produce and export drugs to countries without adequate manufacturing capacity, in return for a pledge from countries not to use the Decision "...to pursue industrial or commercial policy objectives"[16]. Eligible importing countries include least-developed countries or a country that can demonstrate insufficient or no manufacturing capacity for the purpose of meeting its needs. No country, at the time of writing, has yet notified the WTO of its intention to operate as an importer under this decision. Countries may in fact be reluctant to do so as a result of economic and political pressure.

### Ghana's Patent Law

The Patent Law of 1992 provided for the protection of patents in Ghana until recently. This law provided that all inventions, products or processes which were "new, involve an inventive step and are industrially applicable," were patentable (Sec.2)[17]. Pharmaceuticals were considered patentable inventions and patent duration was 10 years (Sec.31(1)). The law permitted compulsory licensing in cases of no or insufficient local working of the patented invention (Sec.45(1)), based on the interdependence of patents (Sec.46), and for products or processes declared to be of vital importance to defence, economic or public health interests (Sec.47). Section 30 considered the patent holder's right exhausted only when he put his patented product on the Ghanaian market, rendering parallel importation impossible. To meet all TRIPS obligations and take advantage of its safeguards, the Ghanaian government reviewed the Patent Law of 1992 and passed a bill to replace it in early 2003.

The changes introduced in the 2003 Patent Act removed some legal tools that may have helped improve access to medicines[18]. Under Section 7 of the 1992 Patent Law, the Ghanaian government had the authority to temporarily exclude inventions or discoveries, such as pharmaceuticals, from patentability "... in the interests of national security, economy, health or any other national concern." The 2003 Patent Act removed this exception. Arguably, the government of Ghana could have excluded specific pharmaceutical products from patentability as a temporary means to address urgent public health concerns. Temporary excludability is particularly useful when procedural requirements to compulsory licensing cannot be met[9]. However, as Correa explains, a literal interpretation of Article 27.1 does not allow the exclusion of pharmaceuticals[9]. He notes that under TRIPS Article 27.2 *ordre public *and Article 8.1 "...pharmaceuticals might conceivably be excluded from patentability, but neither appear sufficient to justify this exclusion except in limited circumstances,"[9]. In any case, the option of using temporary excludability appears unviable at the present time.

Section 13(2) addresses the royalty rate for compulsory licenses: "... [t]he exploitation of the invention...shall be subject to the payment to the owner of an adequate remuneration, taking into account the economic value of the Minister's decision as determined in the decision..."[18]. Adequate remuneration is undefined, allowing for the negotiation of prices, which could have either positive or negative effects on price control and access to medicines. The Ghanaian government has since developed administrative guidelines, proposing the creation of a committee that would determine the level of compensation to be given to a patent holder. It proposed that remuneration for drugs used to treat HIV/AIDS, TB and malaria not exceed 1% of the retail price. In Canada, a country with extensive history of compulsory licensing, a royalty rate of 4% was used[19]. Furthermore, Canada's recent *Jean Chrétien Pledge to Africa Act *may offer useful guidance, as the royalty rate varies from 4 to 0.02% depending upon the importing country's standing on the UN Human Development Index[20].

Section 12(1) of the 2003 Patent Act incorporated Article 33 of TRIPS, doubling the period of patent protection to 20 years. The result will be a delay in the entry of generic competition, and since generic competition tends to lower drug prices, a reduction in overall cost-savings is likely[21]. This provision does not offer any flexibility to member countries; therefore the ensuing barriers will require circumvention via other TRIPS safeguards or policy alternatives. As noted earlier, the Ghana's major disease burden includes malaria, TB and HIV/AIDS. While these diseases can be treated with off-patent medications, extended patent life will be problematic in situations where no other therapeutic options are available[22]. Specifically, evidence of resistance to traditional antimalarial therapy (*e.g*., chloroquine) exists and patients who develop resistance to anti-retroviral medications or experience treatment failure will need access to new, patented medicines in the future. The increase in duration of patent protection impedes Ghana's autonomy over defining their population's therapeutic needs.

Changes were also introduced in the 2003 Patent Act that may promote access to medicines. Section 11(4a) of the 2003 Patent Act allows the international exhaustion of intellectual property rights. This legalizes the parallel importation of lower-priced pharmaceuticals from other countries into Ghana, which will be discussed in detail below. Compulsory licenses can now be issued in circumstances of anti-competitive practice, which allows Ghana to remedy abusive practices and excessive prices, potentially increasing the availability of affordable medicines[9]. Potential also exists to use TRIPS' anti-competitive provisions, accompanied by appropriate national competition policy, to promote the development of the local pharmaceutical industry[8]. The successful suit by the Aids Law Project (ALP) against two major pharmaceutical companies with the South African Competition Commission in 2002 illustrates this potential. Other positive changes include new procedures in granting compulsory licenses: a waiver to seek a voluntary license in "cases of national emergency or other circumstances of extreme urgency," (Section 13(10)) and ministerial authorization instead of the previous lengthy and resource-intensive requirement of legislative authorization (Sec.13(1)).

The 2003 Patent Act widens the provision for issuing a non-voluntary license under local working requirements. Local working requires the manufacturing of a patented product to a minimum degree within the country, potentially stimulating growth of the local pharmaceutical industry. Specifically, the 2003 Patent Act allows non-voluntary licenses in situations where "...the patented invention is not exploited or is insufficiently exploited by working the invention locally, or by importation in the country," (Sec.14(1)) [18]. Whereas the 1992 Patent Act listed four relatively specific instances, where the compulsory license could be invoked if the invention was not being worked, the 2003 Patent Act makes this more open-ended. The limits of this new clause will likely be drawn by the TRIPS agreement and legislative intent during drafting the Patent Bill in Ghanaian courts; its exact interpretation is still unclear. Given the USTR complaint against Brazil regarding its local working requirement, however, the current political feasibility of including and invoking such a clause is tenuous[19].

### How will these developments impact access to medicines in Ghana?

#### Compulsory Licensing

Ghana's 2003 Patent Act, TRIPS and the Doha Declaration offer Ghana sufficient legal ground to use compulsory licensing to address its public health concerns. Compulsory licensing can be used for either local pharmaceutical production or importation, however the latter may be more feasible in the short-term. This will be discussed below. Paragraph 5b of the Declaration explicitly reaffirms the right of countries to "...grant compulsory licenses and the freedom to determine the grounds upon which such licenses are granted." As Correa notes, Paragraph 5b merely states the obvious: Article 31 of TRIPS only requires certain conditions for the granting of compulsory licenses, "but it does not limit the grounds on which such licenses can be granted"[12]. Paragraph 5(c) further facilitates compulsory licensing through the recognition that "public health crises ... can represent a national emergency or other circumstance of extreme urgency" and clarifies that members need not declare a "fully-fledged national emergency"[23].

From a political perspective, the feasibility of using compulsory licensing to address public health concerns has also become more favourable. One event suggesting this is the abandonment of a pharmaceutical industry lawsuit seeking to remove South Africa's amendment in its Medicines and Related Substances Act, which would permit compulsory licensing and parallel importation. The suit was abandoned due to international pressure and the resolve of the South African government. Other events include Brazil's successful use of the threat of compulsory licensing in negotiations to obtain significant discounts of 40–65% on patented antiretrovirals from Roche and Merck and a public statement by Boehringer-Engelheim in 2003 that it will not interfere in the issuance of compulsory licenses and will respect the Doha Declaration[24]. The World Health Organization (WHO) also explicitly supports developing countries in the use of TRIPS safeguards to promote access to medicines[25]. Given these developments, political and legal repercussions from other powerful countries are less likely. For a country to make effective use of compulsory licensing a number of other potential barriers must be addressed and certain requirements must be met.

Effective implementation of compulsory licensing requires the adequate know-how and administrative infrastructure, however many developing countries, including Ghana, do not have this requisite capacity[26]. Article 67 of the TRIPS Agreement requires developed countries to provide technical assistance, "on request and on mutually agreed terms and conditions", to developing and least-developed countries to help address such gaps. Developing countries like Ghana should use this provision and approach developed countries and international organizations for support.

The usefulness of compulsory licensing, for local production or as a negotiating tool, largely depends on whether the appropriate technological and production capacity exists and whether appropriate human resources are available. The experience of Brazil provides an illustrative example on this point. Cohen and Lybecker argue that Brazil's success is based on three main factors: first, the threat posed by Brazil is credible in that it has a viable local industry; second, Brazil's market is "...one of the largest in Latin America and among the top ten globally;" third, Brazil initiated its threats with the strong support of the international community[24]. As we noted earlier, the international community continues to be supportive of developing countries' use of compulsory licensing to address public health needs. In comparison to Brazil, however, Ghana's pharmaceutical market is small which may make the process economically unfeasible. The viability of Ghana's local industry is also questionable as approximately 30 pharmaceutical manufacturing facilities exist but only 17–18 produce throughout the entire year. Careful cost-benefit analysis of the value of domestic production versus the importation of pharmaceuticals is necessary to determine whether Ghana can benefit from compulsory licensing for local production, as it is commonly more economical to import medicines than to produce them locally.

#### Parallel Importation

Parallel imports are of particular importance in meeting public health needs since the pharmaceutical industry generally sets differential prices globally for the same medicine. Thus, parallel importation of a patented medicine from a country where it is sold at a lower price will enable more patients in the importing country to gain access to cheaper drugs (whether international exhaustion applies to medicines produced under compulsory licensing, however, is still a live debate; see Abbott 2002). Paragraph 5(d) of the Declaration explicitly reaffirms members' freedom to determine their own regimes for the exhaustion of intellectual property rights without challenge. Ghana's 2003 Patent Act finally facilitates this by incorporating this provision; it allows parallel importation only under the condition that the product to be imported is already "put on the market in any country by the owner of the patent or with the owner's consent," (Sec.11(4a)).

Parallel importing introduces more challenges. Administrative capacity issues exist with parallel importation as with compulsory licensing. In Ghana, import permits for companies engaged in the parallel importation of drugs are difficult to obtain at this time, which may pose another barrier. Parallel importation increases the opportunity for the influx of sub-standard products and thus attendant recall problems. Some critics argue that parallel importing acts as a disincentive to differential pricing by research-based pharmaceutical companies due to a risk of diversion of low-cost products to lucrative, developed country markets; however, as Outterson notes "...empirical evidence to date does not indicate a sizable arbitrage market in ARVs from low income countries into the high income countries"[20]. Furthermore, there have been no reported cases of diverted drugs from Ghana to other markets. The European Commission's (EC) May 2003 Regulation to facilitate differential pricing may provide another option while lessening some of the industry's re-exportation concerns. The EC regulation provides anti-diversion measures against specific pharmaceutical products and requires manufacturers to reduce their essential medicines export prices to developing countries by "75% off the average 'ex factory' price in OECD countries, or at the cost of production plus 15%"[27]. If parallel importation is to be useful to Ghana, administrative, institutional and managerial capacity must be developed for effective implementation, to prevent the unlawful importation and exportation of products and to ensure quality control.

#### Importation Pursuant to Paragraph 6

If Ghana decides against using its' local industry for the production of generic medicines, Paragraph 6 may offer another potential solution. While some critics have viewed this provision favourably, others have criticized it as too administratively complex for developing countries. Correa explains that the implementation of the Paragraph 6 Decision requires a number of steps, among which include: 1) in most cases, compulsory licenses issued by importing and exporting countries, 2) the importing country's establishment of insufficient or no local manufacturing capacity in the specified pharmaceutical sector, 3) importer notification to the WTO of its intention to use the system detailing product(s) requested and quantities (accompanied by confirmation of insufficient manufacturing capacity and that a compulsory license is or will be granted), and 4) notification of the exporting country's compulsory license to the WTO and the conditions attached[28]. Paragraph 4 of the Decision requires the importing country to "take reasonable measures within their means ... to prevent re-exportation"; this requires countries to implement anti-diversion measures including special marking and labelling of the product(s)[15]. An obstacle might be introduced with strict data protections laws, as exporters must either obtain authorization from the patent holder to obtain efficacy and toxicity data or when denied, perform its own clinical studies to collect this data; this can increase the exporter's costs, therefore increasing the drug cost in the importing country[28]. Additional obstacles and delays may depend on the importing country's national laws on product registration and the exporting company's capacity to manufacture the specified product[28]. Lastly, the Decision limits the exporting country's compulsory license to a "single-supply basis," implying that this entire process must repeat for each new request[28].

Clearly, administrative barriers may hinder some developing countries from using this; however, Article 67 may be used to obtain external assistance to overcome these gaps. Some critics have also claimed that the Decision will not benefit smaller-sized markets, providing minimal incentive to exporting manufacturers. Paragraph 6 of the Decision may help alleviate this problem. It waives the obligations of TRIPS article 31(f) "to the extent necessary to enable a pharmaceutical product produced or imported under a compulsory license in that member to be exported" to other developing or LDCs that are party to the same regional trade agreement and share the health problem in question. Theoretically, this allows a country like Ghana to harness economies of scale and generate more incentive for generic manufacturers to export. However, such a regional trade area must have been formed in conformity with the provisions of Article XXIV of GATT[15].

To date, only Canada, Norway and the Netherlands have passed legislation to allow export of pharmaceuticals under this provision[29,30]. To implement Paragraph 6, developing countries will have to address barriers introduced by these exporting countries. For instance, the recently passed Canadian bill contains a restrictive list of medicines that can be produced for the purposes of Paragraph 6. Norway's legislative counterpart is less restrictive. Whether exporting countries will be able to satisfy the global demand for affordable, generic drugs will be observed.

It is also unclear whether a developing country like Ghana can use Paragraph 6 and if so, under what circumstances. LDCs are automatically eligible, but since Ghana is a developing country, it is required to examine its local manufacturing capacity and establish that "...excluding any capacity owned or controlled by the patent owner, it is currently insufficient for the purposes of meeting its needs"[15]. Ghana has been classified as a country that can reproduce drugs as long as it imports the active ingredients, therefore a cost-benefit analysis is necessary to determine whether or not local manufacture is both technically and economically viable[10]. Whether the lack of economic viability would be considered as "insufficient local manufacturing capacity" in the event of a dispute is questionable. Correa argues that if low-priced medicines cannot be produced because "...meaningful economies of scale have not been reached..." then one of the main objectives of the Doha Declaration, "to promote access to medicines for all," cannot be reached[12]. However, reports indicate that the US informed the Philippines and other countries that it does not consider "economic efficiency" as valid ground for the use of Paragraph 6[31,32]. It is also important to note that the General Council Chairperson's Statement on 30 August 2003, includes a mechanism that allows any member to challenge another member's "interpretation and implementation" of the Decision "with a view to taking appropriate action"[16]. The legal implications of the Chairperson's Statement must still be observed; it is unclear whether a developing country like Ghana will be able to use Paragraph 6 without legal challenge or political and/or economic consequences.

#### Strengthen local industry capacity

The TRIPS Agreement has several provisions, which deal explicitly with the issue of technology transfer. For instance, article 7 states, "The protection and enforcement of intellectual property rights *should contribute to technological innovation and to transfer and disseminate technology*, to the mutual advantage of producers and users of technological knowledge and in a manner conducive to social and economic welfare and to a balance of rights and obligations" [emphasis added]. Despite such provisions, little technology transfer to developing countries has taken place[33]. To strengthen local industry, developing countries like Ghana should still pursue initiatives to absorb new technology. Public-private partnerships (PPPs) may be one mechanism to achieve this. Generally, PPPs require private sector companies to provide the technology and expertise while public sector partners provide development funding and help ensure access to the medications. PPPs can facilitate technology transfer to developing and least-developed countries while creating opportunities to initiate research into developing country diseases[23].

#### Pooled Procurement

Pooled procurement is a cost-containment strategy that can assist developing countries in financing of the drug needs of their populations, as it is the one area of the drug supply management cycle that can offer the greatest amount of cost savings. For example, the Eastern Caribbean Drug Service (ECDS) used pooled procurement to lower pharmaceutical expenditure by an average of 44%[34]. The recently established West Africa Manufacturers Association has put in place mechanisms to take advantage of the economics of scale in the pooled procurement of both raw materials and finished products. In (Economic Community of West African States), political realities have to be addressed given that Francophone West-African countries are already involved in pooled procurement procedures. Thus, if Ghana participates, it would have to comply with established procedures. Ghana must carefully examine the costs and benefits of different procurement policies to determine which ones are most viable and cost-effective. Effective implementation, to be sure, demands institutional capacity, financial management systems, quality assurance and transparency.

#### Voluntary Differential Pricing Arrangements

Ghana can also pursue the procurement of affordable drugs through voluntary differential pricing arrangements. These arrangements can operate through supplier's charity, desire for favourable public relations, or other criteria not immediately or apparently related to market forces. Currently, limited implementation of differential pricing outside of anti-retroviral therapies occurs in Ghana. As noted earlier, the research-based pharmaceutical industry often cites the risk of re-exportation of these drugs to developed country markets as a barrier to scaling up these initiatives, despite the lack of sufficient evidence. To mitigate these concerns, pharmaceuticals firms often require that recipients of their drugs sign a supply agreement that indicates that the recipient will take measures to ensure the security of the drug supply they receive. To further lessen this risk, companies have special packaging and labelling of drugs provided under special programmes like the Accelerated Access Initiative. Developing countries like Ghana can comply with these measures with assistance from firms and established programs to further encourage differential pricing arrangements.

## Conclusion

In this paper, we discuss several possibilities for working within the TRIPS regime to gain better access of the population to medicines. These options include compulsory licensing, parallel importing, technology transfer, local production and voluntary differential pricing. We put forward some favoured policy choices for Ghana. First, we encourage Ghana and its Access to Medicines (ATM) Advisory Committee to consider local production. Local manufacturing can be an effective option if human and technological capacity is scaled up. However, we emphasize that this option should only be pursued if it makes economic sense. As a start, an objective cost-benefit analysis should be done to determine whether it makes economic sense for Ghana to pursue local production. Among the alternatives available to strengthen local industry include more aggressive technology transfer.

Next, we encourage the use of compulsory licensing. If Ghana decides to pursue compulsory licensing, it must then address administrative and knowledge barriers. This can be achieved through obtaining support from developed countries and/or international organizations on the effective implementation of compulsory licensing. There is great potential for Ghana particularly given the Government's commitment to build up its knowledge base in this area. In September 2004, members of the Ghanaian Access to Medicines (ATM) Advisory Committee visited Canada to learn about Canada's past experience with compulsory licensing and what measures could be applied to Ghana. If, however, Ghana determines that it is more technically or economically feasible to pursue importation, Paragraph 6 may provide an option. Ghana will first have to establish insufficient manufacturing capacity in the pharmaceutical sector in question, and then consider what political or economic repercussions may follow. More concrete alternatives for importation include parallel importation or the voluntary tiered-pricing arrangement proposed by the European Commission. Importantly, it is critical to monitor any public policy reform to assess whether or not they are achieving attendant outcomes and adjust accordingly. This will require baseline assessments and regular review at intervals.

The opportunities presented above can only be effective in addressing access to medicines in Ghana if other existing barriers are simultaneously addressed. First and foremost, the development and implementation of an effective exemption policy for the poor without co-payments is vital. Policies can vary such as implementing a national pricing policy that control prices on the supply side by regulating actual drug costs or the demand side, through reimbursement schemes such as reference-based pricing or generic substitution policies. Furthermore, reduction of mark-ups in the public sector may generate competition and drive private sector prices down. A hard but necessary policy reform is needed in the area of national tax, tariff and mark-ups to determine what changes could facilitate more affordable prices for the population.

Is the Ghana case generalisable for other African countries? We hope that as a minimum this case adds to the debate in other African countries about public policies they should pursue to improve access to medicines. Some policies may be more applicable than others depending on economic and political realities. There is not a "one-size-fits-all" policy menu that should be applied. Governments need to make informed policy choices when it comes to improving access to medicines and assess which measures are most needed and viable for their particular country.
